# Phenotype and Genetics of Progressive Sensorineural Hearing Loss (*Snhl1*) in the LXS Set of Recombinant Inbred Strains of Mice

**DOI:** 10.1371/journal.pone.0011459

**Published:** 2010-07-07

**Authors:** Konrad Noben-Trauth, Joseph R. Latoche, Harold R. Neely, Beth Bennett

**Affiliations:** 1 Section on Neurogenetics, Laboratory of Molecular Biology, National Institute on Deafness and Other Communication Disorders, National Institutes of Health, Rockville, Maryland, United States of America; 2 Institute for Behavioral Genetics, University of Colorado, Boulder, Colorado, United States of America; Stanford University School of Medicine, United States of America

## Abstract

Progressive sensorineural hearing loss is the most common form of acquired hearing impairment in the human population. It is also highly prevalent in inbred strains of mice, providing an experimental avenue to systematically map genetic risk factors and to dissect the molecular pathways that orchestrate hearing in peripheral sensory hair cells. Therefore, we ascertained hearing function in the inbred long sleep (ILS) and inbred short sleep (ISS) strains. Using auditory-evoked brain stem response (ABR) and distortion product otoacoustic emission (DPOAE) measurements, we found that ISS mice developed a high-frequency hearing loss at twelve weeks of age that progressed to lower frequencies by 26 weeks of age in the presence of normal endocochlear potentials and unremarkable inner ear histology. ILS mice exhibited milder hearing loss, showing elevated thresholds and reduced DPOAEs at the higher frequencies by 26 weeks of age. To map the genetic variants that underlie this hearing loss we computed ABR thresholds of 63 recombinant inbred stains derived from the ISS and ILS founder strains. A single locus was linked to markers associated with ISS alleles on chromosome 10 with a highly significant logarithm of odds (LOD) score of 15.8. The 2-LOD confidence interval spans ∼4 Megabases located at position 54–60 Mb. This locus, termed *sensorineural hearing loss 1* (*Snhl1*), accounts for approximately 82% of the phenotypic variation. In summary, this study identifies a novel hearing loss locus on chromosome 10 and attests to the prevalence and genetic heterogeneity of progressive hearing loss in common mouse strains.

## Introduction

Hearing loss is one of the most common sensory impairments, the causes of which are many fold and varied. For instance, congenital hearing impairment affects 1 out of 700–1000 newborns [1]. In addition, approximately 15% of Americans between the ages of 20 and 69 suffer from noise-induced hearing loss (NIHL), primarily due to occupational and recreational noise exposure. Furthermore 30% of those between 65 and 74 years of age develop progressive age-related hearing impairment (ARHI, presbycusis) [2].

The genetics of inherited hearing impairment is rather well understood; it is heterogeneous, typically monogenic and caused by rare, highly penetrant, and mostly disabling mutations (http://webh01.ua.ac.be/hhh/). In contrast, the genetic hallmarks, i.e. frequency, penetrance, and complexity of risk alleles of acquired hearing loss are largely unknown. There is little doubt that susceptibility and resistance to non-Mendelian and environmentally induced forms of hearing loss such as NIHL, ARHI, and tinnitus are due to genetic variation [3]–[5]. For example, several twin studies and pedigrees have shown a strong heritability coefficient correlated with presbycusis and a genome-wide association study recently linked a single nucleotide polymorphism in the metabotropic glutamate receptor type 7 (*GRM7*) gene to increased risk of ARHI [6].

The prevalence of sensorineural hearing loss in inbred strains, as well as the conserved molecular genetics between mouse and humans, provides a unique experimental avenue to systematically identify the underlying genetic risk factors of hearing loss [7], [8]. The LXS set comprises 63 recombinant inbred (RI) strains that originated from the parental inbred long sleep (ILS) and inbred short sleep (ISS) mouse strains. The ILS and ISS founder strains were derived from a multi-generation cross of eight laboratory strains [9], which were initiated about 50 years ago. The hearing status of the founder strains is unknown. However, substrains of six (A, AKR, BALB, C57BL, and DBA/2) of the eight founder strains were later shown to have early- or late-onset hearing loss, while the extant C3H/2 and RIII strains were shown to have normal hearing, and the hearing status of the ISBI strain is unknown [10].

We screened this RI panel for auditory function for several reasons. First, given the genetic origin of the founder lines it is conceivable that novel and previously unrecognized hearing loss alleles segregate in this RI set. Second, the panel provides sufficient statistical power (*p*<0.05) to identify quantitative trait loci (QTLs) that account for ∼25% of the genetic variation. Third, the panel incorporates approximately 3600 recombination breakpoints, providing a resolution of 1–10 cM for the mapping of QTLs [11]. Fourth, the entire panel has been genotyped at ∼11,000 SNP marker loci greatly facilitating the genetic linkage analysis. Finally, all genetic and phenotypic data from the LXS RI set are integrated in a web-based analysis platform that includes software for one- and two-dimensional genome-wide regression analyses (www.genenetwork.org). These factors make screening of these inbred strains ideal for determining the genes and molecular mechanisms that contribute to hearing loss.

## Results

### ILS and ISS strains auditory phenotypes

**Figure 1:**
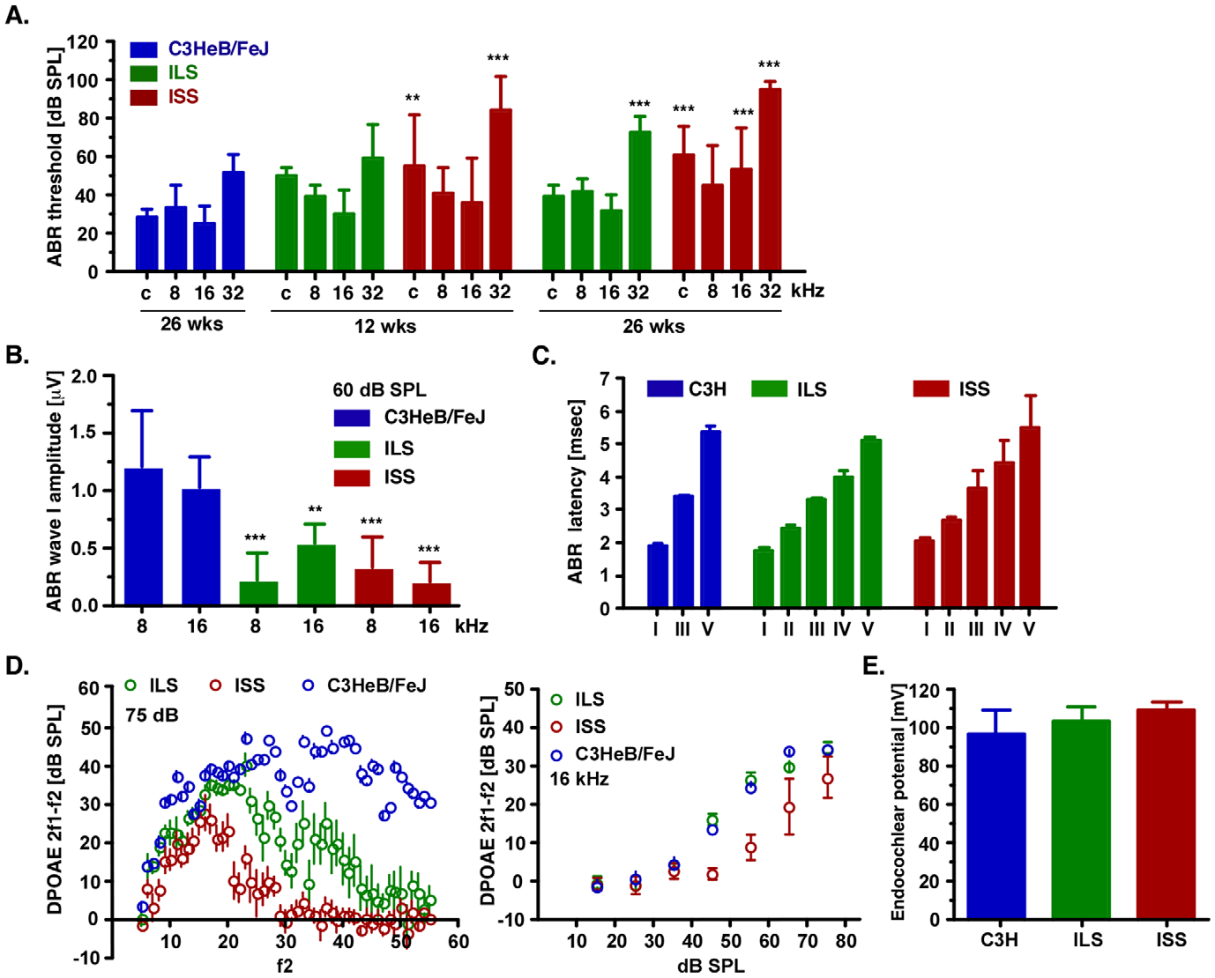
Auditory characteristics of ILS and ISS mice. **A.** ABR thresholds at click (c) and pure tone pips at 8-, 16-, and 32 kHz at twelve and 26 weeks (wks) of age. Data are given as mean ± SD. **B.** Wave I ABR amplitudes at 8- and 16 kHz stimuli at 60 dB SPL input levels. Data are given as mean ± SD. µV, microvolt. **C.** Latencies of ABR waves I – V at a 16 kHz stimulus of 60 dB SPL. Data are given as mean ± SD; msec, milliseconds. **D.** DPOAE output levels at 2f1-f2 in dB SPL over f2 frequency range 6–56 kHz (left panel). The right panel shows I/O function at f2 = 16 kHz. Data are given as mean ± SEM. **E.** Endocochlear potentials. Data are given as mean ± SD; mV, milliVolt. For all panels: C3HeB/FeJ (blue), ILS (green) and ISS (red); **p<0.01; ***p<0.001.

To determine the hearing function of ILS and SS mice, we measured thresholds and wave I amplitudes of auditory-evoked brain stem responses. In twelve-week-old ISS mice we found significantly elevated thresholds at the click (*p*<0.01) and 32 kHz stimuli (*p*<0.001), compared to normal hearing, age-matched C3HeB/FeJ mice. The mean thresholds at the 8- and 16 kHz stimuli were not different although the standard deviation (13- and 23 dB SPL) was significantly higher than in the C3HeB/FeJ controls (*p*<0.001) (**Fig. 1A** **,** **Table 1**). At 26 weeks of age, hearing thresholds in ISS mice were elevated across all tested stimuli (*p*<0.001), being highest at the 32 kHz and lowest at the 8 kHz stimuli. ILS mice showed normal hearing thresholds at twelve weeks of age. However, at 26 weeks there was a noticeable hearing loss at the high frequency 32 kHz stimulus with a mean threshold of 72±9 dB SPL, which was 22 dB SPL higher than that of C3HeB/FeJ controls (*p*<0.001; **Fig. 1A** **,** **Table 1**).

**Table 1:** ABR thresholds in ILS and ISS strains.

		click		8 kHz		16 kHz		32 kHz		n
	wks	mean ± SD	*p*	mean ± SD	*p*	mean ± SD	*p*	mean ± SD	*p*	
**C3H**	26	32±2.6		29±5		22±3		50±10		6
**ILS**	12	50±4.1	n.s.	39.3±6.1	n.s.	30±12.3	n.s.	59.2±17.7	n.s.	7
**ISS**	12	55±26.5	<0.01	41.3±13	n.s.	36.3±23.1	n.s.	84.4±17.8	<0.001	8
**ILS**	26	39.5±5.9	n.s.	41.6±6.9	n.s.	31.6±8.7	n.s.	72.4±8.7	<0.001	19
**ISS**	26	60.8±14.9	<0.001	45±20.8	n.s.	53.3±21.6	<0.001	95±4.5	<0.001	6

C3H, C3HeB/FeJ; wks, weeks of age; kHz, kiloHertz; SD, standard deviation; *p*, ANONVA; n.s., not significant; n, number of animals tested;

To determine whether the compound auditory nerve action potential is affected, we measured the wave I ABR peak-to-peak amplitude at supra-threshold hearing levels (60 dB SPL at 8- and 16 kHz). In both ILS and ISS mice, we observed a significant decrease in amplitudes at 8- and 16 kHz compared to C3HeB/FeJ at 26 weeks of age (*p*<0.001) (**Fig. 1B**). For instance, in both ILS and ISS mice, the amplitude at 8 kHz and 60 dB SPL stimuli measured 0.2±0.2 µV (n = 16) and 0.3±0.2 µV (n = 3), respectively, compared to 1.2±0.5 µV (n = 6) for C3HeB/FeJ control mice. To determine whether neural propagation of the ascending auditory pathway was compromised, we measured the latencies of ABR waves I through V. In 26-week-old ILS and ISS mice, there was no significant difference in latencies at the 8- and 16 kHz stimuli compared to C3HeB/FeJ controls (**Fig. 1C**).

To differentiate the abnormal compound auditory responses, we ascertained the function of the sensory outer hair cells directly by using distortion-product otoacoustic emission (DPOAE) tests. In 26-week-old ISS mice, we found complete absence of distortion-products at L1 = 75 dB SPL and f2 = 30–52 kHz. At f = 18–30 kHz, we measured a significant reduction in emission levels (*p*<0.001), while at the lower frequency range (f2 = 6–16 kHz) DPOAE levels were comparable to control mice (**Fig. 1D**). In 26-week-old ILS mice, we measured a significant reduction in DPOAE levels at f2 = 20–52 kHz, which was most pronounced at the 45–52 kHz range and progressed from higher to lower f2 frequencies (**Fig. 1D**). Emission levels were normal in the lower frequency range in these animals (f2 = 6–20 kHz). To test whether a defect in the stria vascularis could account for the abnormal ABRs and reduced emission levels, we measured the voltage potential in the scala media at the level of the round window. We observed endocochlear potentials (EP) of 104±6 mV (n = 10) and 109±4 mV (n = 4) in 26-week-old ILS and ISS mice, respectively, which was comparable with the potentials measured in normal hearing C3HeB/FeJ mice (94±9 mV; n = 5; *p*>0.05) (**Fig. 1E**).

### Inner ear histology of ILS and ISS mice

**Figure 2:**
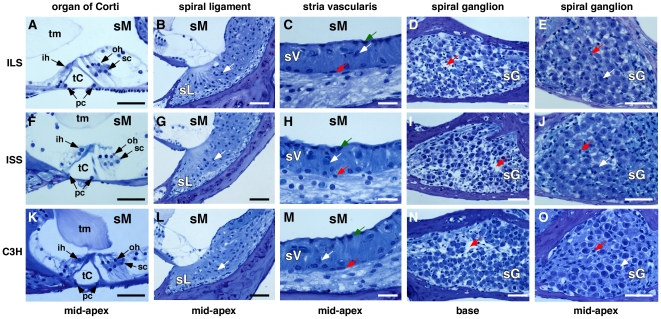
Inner ear histology in ILS and ISS mice. Tolouidin blue-stained plastic sections through the cochlear duct in twelve-week-old ILS and ISS and eight-week-old C3HeB/FeJ (C3H) mice. **A,F,K,** Cross section through the organ of Corti at the mid-apical region; tm, tectorial membrane; oh, outer hair cell; ih, inner hair cell; sc, supporting cell; tC, tunnel of Corti; sM, scala media; scale bar  =  50 µm. **B,G,L,** Cross section through the spiral ligament at the mid-apical region of the cochlear duct. White arrow indicates a fibrocyte. sL, spiral ligament; scale bar  =  50 µm. **C,H,M,** Cross section through the stria vascularis at the mid-apical region of the cochlear duct. Red arrow, basal cell; white arrow, intermediate cell; green arrow, marginal cell; sV, stria vascularis; scale bar  =  10 µm. **D,I,N,** Cross section through the spiral ganglion near the base of the cochlear duct. Red arrow points to areas of degeneration. sG, spiral ganglion; scale bar  =  50 µm. **E,J,O,** Cross section through the spiral ganglion at the mid-apical region of the cochlear duct. White arrow points to a neuron and the red arrow indicates a Schwann cell. sG, spiral ganglion; scale bar  =  50 µm.

To reveal the inner ear histopathology, we obtained modiolar sections of twelve-week-old ILS and ISS cochleae. The organ of Corti (**Fig. 2A, F, K**) and spiral ligament (**Fig. 2B, G, L**), which are commonly affected by progressive hearing loss, were normal in appearance as was the stria vascularis (**Fig. 2C, H, M**). Spiral ganglia at the base showed signs of degeneration in both ILS and ISS ears, but similar slight degeneration was also observed in C3HeB/FeJ controls (**Fig. 2D, I, N**). Neuronal cell counts per 800 µm^2^ section (n = 6) were not significantly different in ILS (35±10) and ISS (30±13) animals compared when to C3HeB/FeJ controls (46±13; *p*>0.05). Furthermore, cell densities in the ganglia at mid-apical regions were also indistinguishable among ILS, ISS and C3HeB/FeJ controls (**Fig. 2E, J, O**).

### Genetic linkage analyses in the LXS RI set

**Figure 3:**
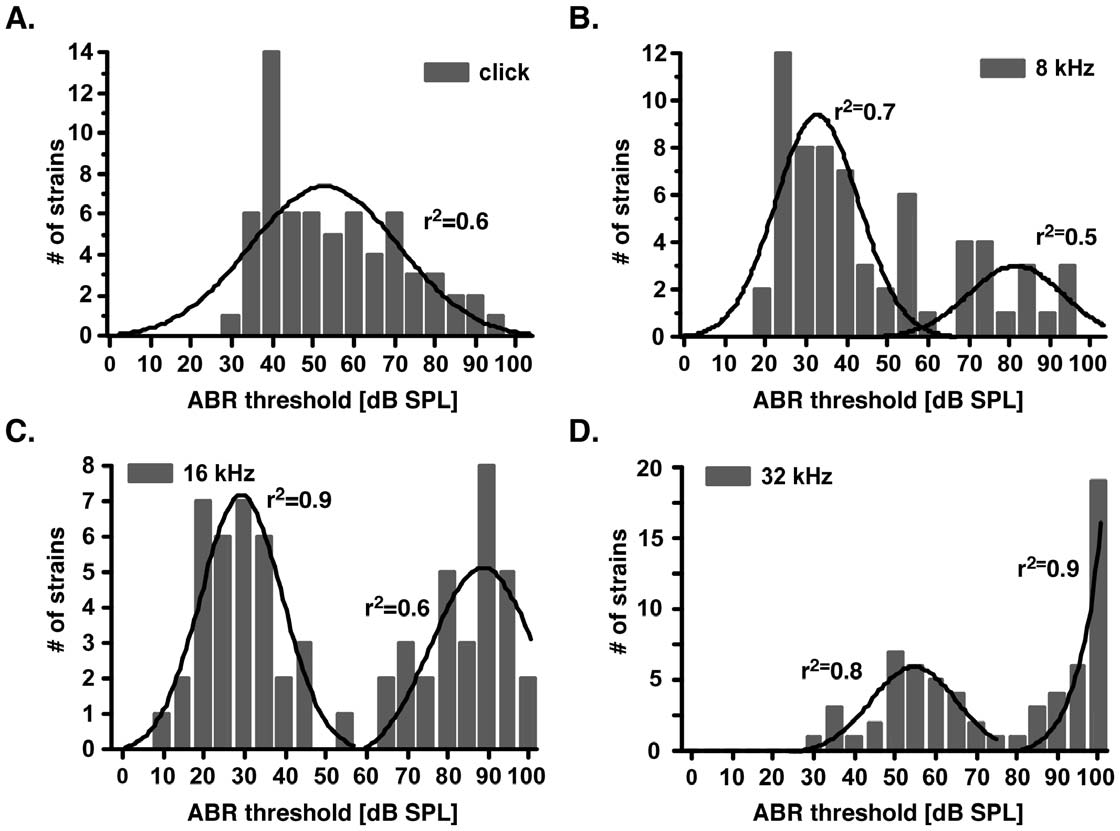
ABR threshold distribution in the LXS RI set. **A–D.** Histograms showing threshold distributions at the click (**A**), 8- (**B**), 16- (**C**), and 32 kHz (**D**) stimuli. The Y-axis represents the number of RI strains, and the X-axis denotes the threshold level. Histograms were fitted with a normal Gaussian distribution. r^2^  =  goodness-of-fit.

To genetically map the loci underlying the hearing impairment in the ILS and ISS mice, we measured ABR thresholds in the 63 recombinant inbred strains derived from the ILS and ISS parental strains. We tested four twelve-week-old mice from each strain using four stimuli. At the click stimulus, thresholds followed a normal distribution, with a mean of 55±17 dB SPL (n = 63). At the 16 kHz stimulus, thresholds followed a bimodal distribution, with means of 28±10 dB SPL (n = 33) and 84±10 dB SPL (n = 30). Thresholds at the 8 kHz stimulus showed a similar bimodal distribution. At the 32 kHz stimulus, threshold values were also distributed bimodally, but the histogram was shifted toward higher mean threshold values of 53±11 (n = 31) and 95±6 dB SPL (n = 32) (**Fig. 3A–D**). These distributions suggested the segregation of one major locus with a dynamic phenotype at twelve weeks of age.

**Figure 4:**
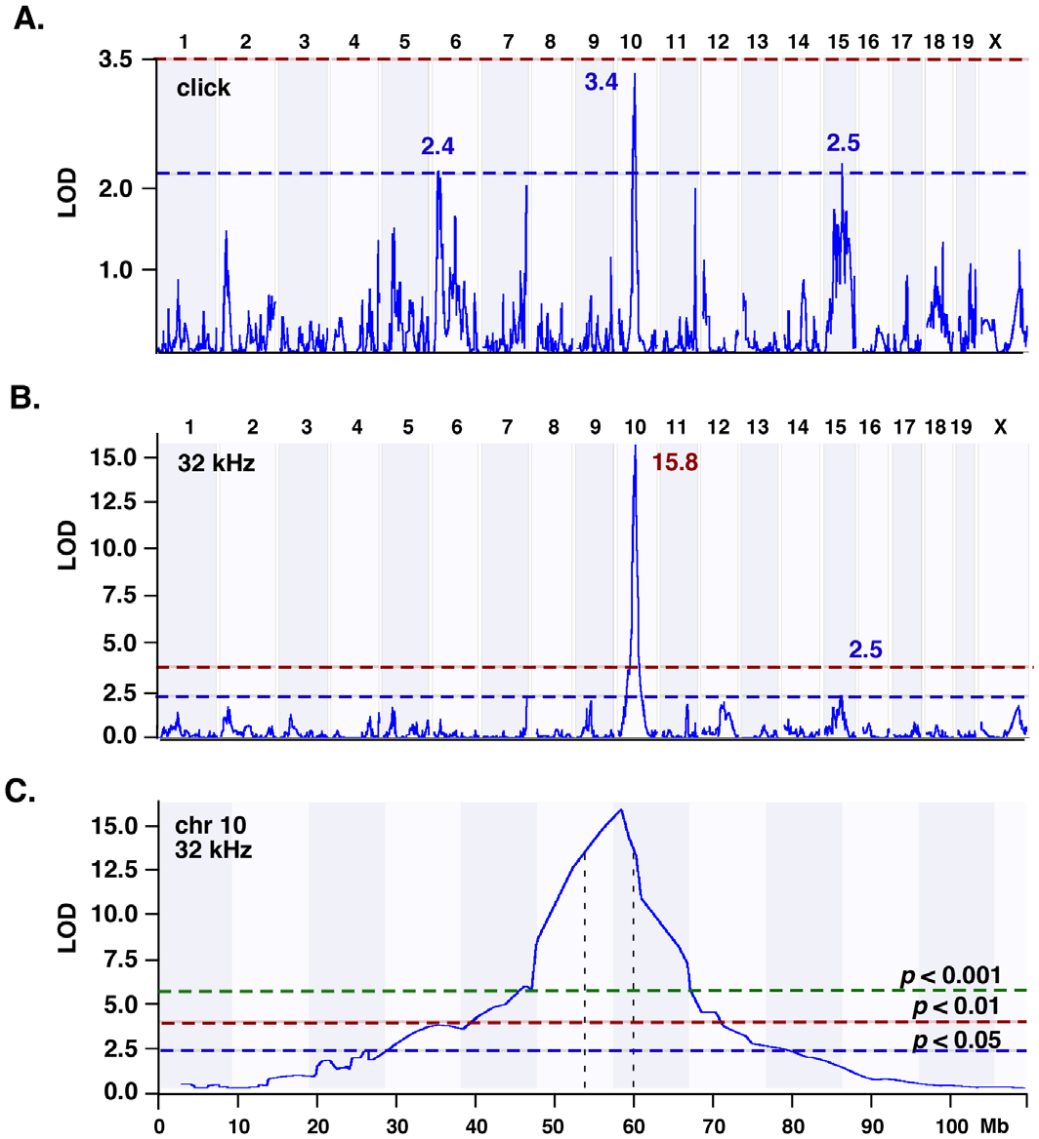
Genome-wide linkage scan. Shown are WebQTL plots of genome-wide (**A, B**) and chromosome-specific (**C**) regression analyses. **A, B.** The X-axis represents the number of chromosome number and the Y-axis gives the LOD score. Red and blue dotted lines indicate the 0.01 and 0.05 genome-wide significance level obtained through permutation testing. Peak LOD scores at chromosome 6, 10 and 15 at the click (**A**) and chromosome 10 and 15 at the 32 kHz (**B**) stimulus are indicated. **C.** Results of chromosome 10 interval mapping. X-axis indicates the physical position in Megabases (Mb) and the Y-axis denotes the LOD score for the thresholds at the 32 kHz stimulus. Dotted horizontal lines indicate the genome-wide significance level. Vertical black dotted line denotes the 2-LOD confidence interval of *Snhl1* at chromosome (chr) 10.

We next performed a genome-wide linkage scan using WebQTL, which employs a linear regression model to compute marker/phenotype correlations. We found highly significant correlations between elevated thresholds at the 8-, 16-, and 32 kHz stimuli and markers on chromosome 10 (*rs3682060*, *rs13480620*; *p*<0.001). The correlation was highest at the 32 kHz stimulus with a logarithm of odds (LOD) score of 15.8 and was associated with ISS-derived alleles. The association between click-derived thresholds and markers on chromosome 10 was only marginal, and was below the genome-wide significance level (*p*>0.01). Additional spurious linkage was obtained with regions on chromosomes 2, 6, 11, 12, and 15 (0.05>*p*>0.01) (**Table 2**; **Fig. 4A, B**). To detect QTLs hidden by the strong locus on chromosome 10, we performed composite QTL mapping. However, this did not reveal any additional significant hits. Interval mapping combined with bootstrap testing and a 2-LOD cut-off margin localized the QTL confidence interval to a ∼4 Mb region localized at the 54–60 Mb position on chromosome 10 (**Fig. 4C**). We named this QTL *Sensorineural hearing loss 1* (*Snhl1*).

**Table 2:** Results of genome-wide regression analysis.

Stimulus	Marker	Chr	LOD	Allele	*p*	2-LOD interval
**click**	*rs3663703*	6	2.4	ISS	<0.05	8–40 Mb
	*rs3682060*	10	3.4	ISS	<0.05	48–61 Mb
	*CEL-15_58115663*	15	2.5	ISS	<0.05	n.d.
**8 kHz**	*rs6407520*	2	2.6	ILS	<0.05	8–52 Mb
	*rs3682060*	10	5.2	ISS	<0.001	48–64 Mb
	*rs3724750*	11	2.8	ISS	<0.05	60–92 Mb
**16 kHz**	*D2Mit81*	2	2.5	ILS	<0.05	4–52 Mb
	*rs13480620*	10	11.7	ISS	<0.001	52–62 Mb
	*rs6335879*	12	2.7	ILS	<0.05	58–108 Mb
**32 kHz**	*rs3682060*	10	15.8	ISS	<0.001	56–60 Mb
	*rs13482581*	15	2.5	ISS	<0.05	28–80 Mb

**Figure 5:**
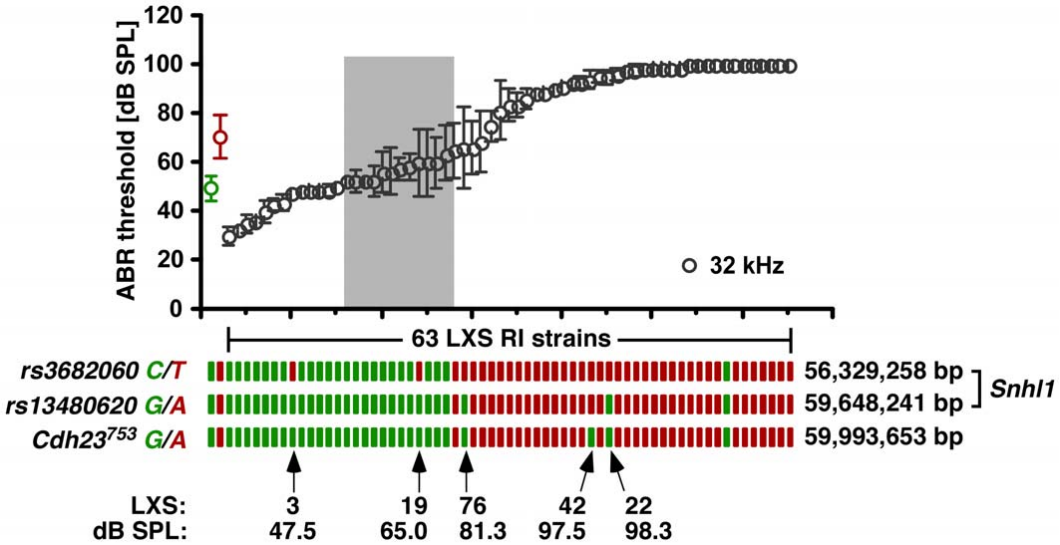
*Snhl1* phenotype/genotype correlation. ABR thresholds at the 32 kHz stimulus of each of the 63 RI strains (grey) and the parental ILS (green) and ISS (red) strains as a function of their genotype at markers *rs3682060*, *rs13480620*, and *Cdh23^753^* are shown. ABR thresholds (in dB SPL; Y-axis) are given as mean ± SD (n = 4). The grey box denotes the number of strains and threshold range unexplained by *Snhl1*. The ISS allele is shown in green (C, cytosine; G, guanine) and the ILS allele is represented in red (T, thymidine; A, adenine) boxes. The number of the RI strain carrying a recombinant chromosome and the threshold at the 32 kHz is shown below the haplotypes. On the right, the physical location of the marker on chromosome 10 in base pairs (bp) is given and the most likely location of *Snhl1* is indicated.

kHz, kiloHertz; QTL, Quantitative trait locus; Chr, chromosome;

To detect interacting QTLs, we performed a two-dimensional genome-wide linkage scan, but no genetic interactions or epistatic effects were measured. We estimate that the *Snhl1* locus accounts for approximately 82% of the phenotypic variation (**Fig. 5**).

To determine the strain origin of the *Snhl1* locus, we compared the haplotypes of ISS and ILS mice with the founder strains over a 10 Mb region (chr10: 51,000,000–61,000,000 bp). Within this haplotype block and among these ten strains, the *C*-allele at marker *rs3682060* is present in ILS and C3H/HeJ strains only, whereas the *A*-allele at *rs13480620* is present in the ISS and DBA/2J strains.

The 2-LOD confidence interval of *Snhl1* contains cadherin-23 (*Cdh23*). A 753G>A polymorphism in *Cdh23* (chr10: 59,993,653 bp) has previously been implicated in age-related hearing loss in C57BL/6J, DBA/2J, and other inbred strains [12]. To ascertain the genetic location of *Cdh23* relative to *Snhl1*, we determined the allele status of *Cdh23^753^* in the parental and the 63 LXS RI strains. Consistent with the origin of the haplotype blocks, we found that mice of the ILS strain carry the *Cdh23^753G^* allele and mice of the ISS strain have the *Cdh23^753A^* allele. Among the 63 RI strains, the *Cdh23^753^* polymorphism was concordant with marker *rs13480620*, except for strain LXS42, which carried the 753G allele in the presence of a profound hearing loss (**Fig. 5**).

## Discussion

In this study, we defined the auditory hallmarks of hearing impairment in the ILS and ISS inbred mouse strains and found delayed-onset sensorineural hearing loss as evidenced by increased ABR thresholds, reduced ABR wave I amplitudes, and absent DPOAEs in the presence of normal endocochlear potentials, unaltered ABR wave latencies and unremarkable inner ear histology. This hearing loss was more pronounced in the ISS strain.

Sensorineural hearing loss affects the organ of Corti and the ascending nerve fibers of the 8^th^ cranial nerve with its associated first order type I and type II neurons. A hallmark of this type of hearing loss is the increased thresholds at higher frequencies. In the present study, thresholds were highest at the 32 kHz stimulus and reduced at the lower frequencies. Furthermore, the ABR threshold progression in the ILS and ISS strains at twelve and 26 weeks of age followed this pattern, and the same succession was detected using DPOAE tests, which revealed a systematic decay in emissions from the higher to the lower frequency spectrum. While diminished distortion-products can also result from a reduced or absent voltage potential in the endolymph, the normal EPs found in ILS and ISS mice argue in favor of a primary defect at the level of the sensory hair cells. The wave I amplitude is the neuronal response of the distal portion of the type I and type II afferent neurons. Approximately 95% of the afferents are type I cells that make contact with inner hair cells, whereas the remainder contact outer hair cells. The reduced wave I amplitudes in ILS and ISS mice may point to a defect in the receptor potential, the size of the synaptic transmission, or levels of the excitatory post-synaptic potentials (EPSPs). In contrast, the conduction velocities and time constants of both EPSPs and synaptic transmission seem to be intact, as suggested by the normal latency of wave I.

The *Snhl1* locus has a strong effect, accounting for 82% of the trait distribution but it leaves the threshold variation of twelve RI strains unexplained. To account for these strains, we performed composite mapping, which masks the *Snhl1* QTL, and interrogated the genome for additional genetic and epistatic interactions. However, no additional genetic effects were observed. The data suggests the segregation of a second QTL that may associate with ILS-derived alleles, which is a reasonable assumption, considering the presence of a milder form of hearing loss in that strain. This hypothesis could be tested by analyzing a segregating cross between ILS and ISS, which would also aid in the fine mapping of *Snhl1*. This second locus may be represented in the intervals on chromosome 2 or 12 that reached only suggestive levels of genome-wide significance and escaped detection due to the resolution-limit of the RI set [11].

The 2-LOD *Snhl1* confidence interval contains 30 genes including *Cdh23*, which is involved in various forms of hearing loss in both mouse and human. Although it is possible that the *Cdh23^753A^* allele or a new variant of *Cdh23* underlies *Snhl1*, three sets of experiments seem to argue against it. First, by haplotype analysis and genotyping of the 63 RI strains at the *Cdh23^753A/G^* polymorphism we identified three RI strains (LXS19, LXS22, and LXS76) with recombinations that place *Snhl1* close to marker *rs3682060*, located 3.7 Mb proximal to *Cdh23*. In addition, RI strain LXS42 caries a recombination that places *Snhl1* outside of the *Cdh23* region and close to *rs13480620*, located 345 kb proximal to *Cdh23^753^*. Second, we sequenced all but two exons of *Cdh23*, but found only one non-synonymous nucleotide change. This variant, Leu5Pro, is also present in the CAST/Ei strain, which exhibits a rather robust hearing function excluding it as the causative variant. Two other variants, also in exon 1, were silent third-position transitions. Third, recombination events in four RI strains (LXS3, LXS19, LXS22, and LXS76) localize *Snhl1* most likely to a 3.3 Mb region delimited by markers *rs3682060* (chr10: 56,329,258 bp) and *rs13480620* (chr10: 59,648,241 bp).

We also compared the haplotype structure of the ILS and ISS strains with the eight founder strains and found that the ISS haplotype block over the *Snhl1* interval is most likely derived from the DBA strain. As both DBA/1J and DBA/2J show progressive and complex hearing loss, this suggests that the *Snhl1* locus may represent one of the DBA hearing loss alleles. Alternatively, *Snhl1* may constitute a new mutation that occurred during the development of the ISS strain.

**Figure 6:**
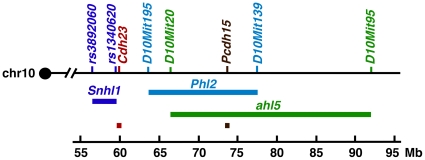
Location of hearing loss QTLs on chromosome 10. Graphic representation of loci involved in hearing function on mouse chromosome (chr) 10. The markers flanking each QTL (*Snhl1*, *Phl2*, and *ahl5*) are given on the top, the 2-LOD confidence interval defined by the markers is given in the middle, and the physical scale (Mb, megabases from the centromere) is given on the bottom.

Another QTL underlying progressive hearing loss, *progressive hearing loss 2* (*Phl2*), was mapped in the 101/H strain [13]. The QTL association peaked with marker *D10Mit115* (chr10: 69,739,444Mb) and the 2-LOD confidence interval, encompassing approximately 17 Mb, ranges from *Cdh23* to *D10Mit139* (chr10:60,091,228–77,695,405 Mb). This QTL interval seems to place *Phl2* outside of the *Snhl1* interval (**Fig. 6**). Furthermore, the 101/H strain is closely related to the group of 129 strains [14]. Given the genetic distance between the 129 strains and the eight founder strains, it seems unlikely that *Phl2* and *Snhl1* are identical or represent allelic forms of the same locus.

Progressive sensorineural progressive hearing loss is a common phenotype in inbred and heterogeneous mouse strains [10], [15], [16]. Previous genetic linkage studies in a subset of these strains demonstrated that it is caused by the segregation of a small number (1–3) of loci per strain that show various types of interaction and inheritance due to epistatic, additive or co-dominant effects [13], [15], [17]–[20]. The ISS strain seems to fit into this paradigm, having one major locus and perhaps one minor player underlying delayed-onset, progressive sensorineural hearing loss.

The molecular physiology of the inner ear, and the genetics of monogenic hearing loss, are highly conserved between human and mouse, suggesting that this genetic conservation also holds up at the level of complex hearing loss, which is thought to underlie the most common forms of acquired progressive hearing impairment, ARHI and NIHL [3], [7], [21]. Under this assumption, the data presented here together with previous results [19], suggest that susceptibility to progressive sensorineural hearing loss in the human population is very heterogeneous, but controlled by a small number of rare and hypomorphic risk alleles on an individual basis.

## Materials and Methods

### Animals

The generation of the LXS panel of RI strains was described elsewhere [11]. Institutional review boards at the National Institutes of Health and University of Colorado approved the animal studies.

### Auditory-evoked brain stem response measurements

Hearing function was assessed using auditory-evoked brain stem response (ABR) measurements controlled by a computer-aided evoked potential system (Intelligent Hearing System, IHS; Miami, Florida). The Smart-EP version 10, modified for high frequency capability and coupled to high frequency transducers was used to generate specific acoustic stimuli, and to amplify, measure, and display the evoked brainstem responses of anesthetized mice. Subdermal needle electrodes were inserted at the vertex (active), ventrolaterally to the right ear (reference) and the left ear (ground). Specific acoustic stimuli were delivered to the outer ear canal through a plastic tube channeled from the high frequency transducers. Mice were presented with click stimuli and with 8-, 16-, and 32 kHz tone pips at varying intensity, from low to high (10–100 dB SPL) at a rate of 19.1 times/sec for a total of 350 sweeps. Sound pressure level thresholds were determined for each stimulus frequency by identifying the lowest intensity producing a recognizable ABR pattern on the computer screen (at least two consistent characteristic wave forms). Peak-to-peak amplitudes and wave latencies were determined using IHS software.

### Distortion-product-otoacoustic emission measurements (DPOAEs)

DPOAEs were measured using National Instruments (NI) LabView 8.6 software, operating an NI PCI-4461 Dynamic Signal Analyzer (DSA) sound card, to generate two pure tones, f1 and f2, at the fixed f2/f1 ratio of 1.3, which were emitted separately by two Clarion SRU310H high frequency dome tweeters placed in the outer ear canal at the presentation level of f2  =  f1- 10 dB  =  50 dB SPL (Sound Pressure Level). The f1 and f2 components were swept in 1 kHz steps starting from f1  =  5 kHz to 55 kHz with f2 = f1x1.3. Intensity levels sweeps ranged from 15 dB SPL up to 75 dB SPL, in 10 dB increments. Sound pressure levels were measured using an Etymõtic-ER-10B+ microphone. The amplitude of the 2f1- f2 distortion product was plotted in dB SPL against the f2 frequency where the DP is generated. Clarion speakers and Etymõtic ER-10B+ microphone were calibrated using a 1/4inch microphone 7016 (1/4inch pre amp 4016 and microphone power supply PS9200, AcoPacific). The AcoPacific 1/4inch microphone 7016 was calibrated using a QC-10 Sound Calibrator (Quest Technologies).

### Endocochlear potential measurements

The endocochlear potential (EP) was measured at the round window. Briefly, the tip of a small glass pipette containing a silver/chloride electrode bathed in 0.1M KCl was inserted through the round window into the endolymph using a remote controlled motorized micromanipulator (PPM5000, Piezo World Precision Instrument). The electrode was connected to a Warner Dual Channel Differential Electrometer (HiZ-223), which amplified and routed the voltage difference (subdermal 1M KCl reference electrode) to a PC-controlled data acquisition system (Digidata 1440A, Axon Instruments) using AxoScope software, which displayed the measured output. Data were sampled at a rate of 10 kHz for 60 sec. The glass electrode was prepared using a Sutter Instrument (P-97 Flaming/Brown micropipette puller) and measurements were performed in a bench top faraday cage (TMC; Technical Manufacturing Corporation).

### Histology

For gross morphology on plastic sections, the ear was removed from the temporal bone and the inner ears were dissected in phosphate buffered saline (PBS), perfused with 4% paraformaldehyde and kept in the same fixative at room temperature for at least 12 hours. Specimen were washed twice in PBS and decalcified in 0.1M EDTA pH 8.0 in PBS for three weeks. The Inner ears were then dehydrated with a graded series of ethanol, infiltrated with JB-4 monomer (Polysciences, Inc.), and embedded for mid-modiolar and saggital sections. Serial sections were cut at 4 µm thickness using a tungsten carbide disposable blade on a RM2265 microtome (Leica) and mounted on Superfrost Plus glass slides. Sections were stained with 0.1% Toluidine Blue O, cleared in xylene, imaged on a DM5000B microscope (Leica) and photographed with a DFC500 digital camera (Leica). Image levels were adjusted with Adobe Photoshop software.

### Genetic linkage analyses

WebQTL was used for linear regression analyses, composite and interval mapping and tests for epistatic interactions available at The GeneNetwork (www.genenetwork.org). Genome-wide significance levels were determined by permutation testing (1000 permutations). QTL confidence interval was determined by bootstrap testing.

### Statistical analyses

Unless otherwise indicated groups of data were compared using one-way analysis of variance (ANOVA) followed by Bonferroni post-hoc test to correct for multiple testing. GraphPad Prism 4.0b software was used to perform column statistics and compute *p* values.
